# Signe de mont Fuji: pneumocephalie compressive

**DOI:** 10.11604/pamj.2015.22.310.8306

**Published:** 2015-11-27

**Authors:** Ali Derkaoui, Mohammed khatouf

**Affiliations:** 1 CHU Hassan II, Service de Réanimation Polyvalente, Fès, Maroc; 2 Université Sidi Mohammed Benabdellah, Fès, Maroc

**Keywords:** Hématome sous durale chronique, pneumocéphalie, signe de mont Fuji, Chronic subdural hematoma, pneumocephalus, Mount Fuji sign

## Image en medicine

Mme S.K, opérée en urgence pour hématome sous durale chronique hémisphérique droit, ayant bénéficié d'un drainage par trous de trépans. L’évolution post opératoire été marqué par son aggravation brutale sur le plan neurologique. Le scanner de contrôle a objectivé une pneumocéphalie frontale bilatérale comprimant les deux lobes frontaux rappelant le Signe de mont Fuji. Le signe du Mont-Fuji désigne de l'air sous-dural dans les régions frontales qui sépare et comprime les deux lobes frontaux. Le silhouettage ainsi crée rappelle (selon les radiologues Japonais) celui du Mont Fuji. C'est une complication rare, pouvant engager le pronostic vitale. Elle se voit au décours d'un geste de neurochirurgie avec ouverture de la dure mère, d'un traumatisme crânien, d'une méningite ou dans certaines invasions tumorales. La prise en charge est essentiellement symptomatique associant une oxygénothérapie et des moyens de lutte contre l'hypertension intra crânienne. La chirurgie décompressive peut être indiquée si la compression est importante avec des signes de pré-engagement.

**Figure 1:**
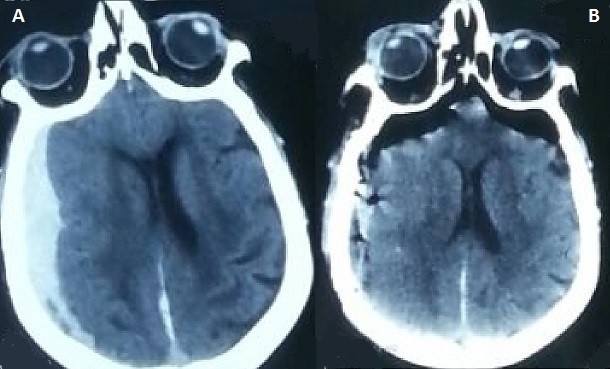
Coupe tomodensitométrique sans injection de produit de contraste; (A) hématome sous dural chronique exerçant un effet de masse sur la ligne médiane (B) pneumocéphalie frontale bilatérale comprimant les deux lobes frontaux: signe de mont Fuji

